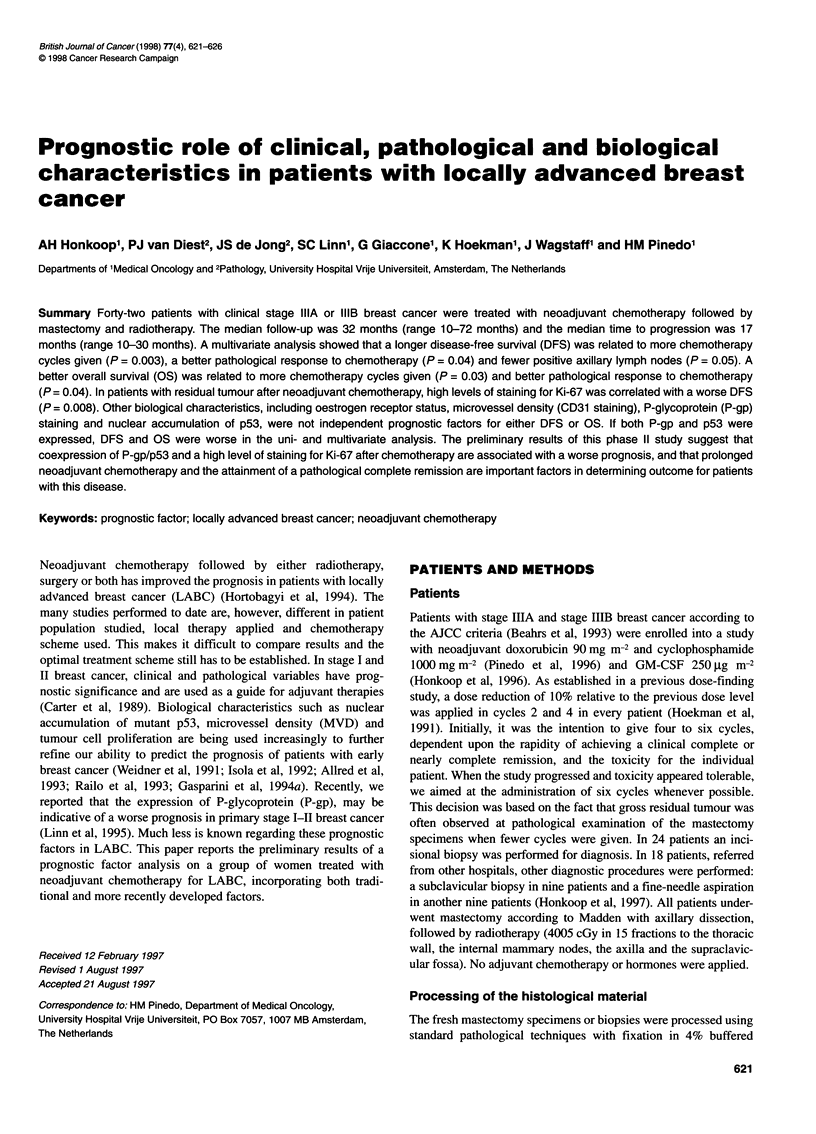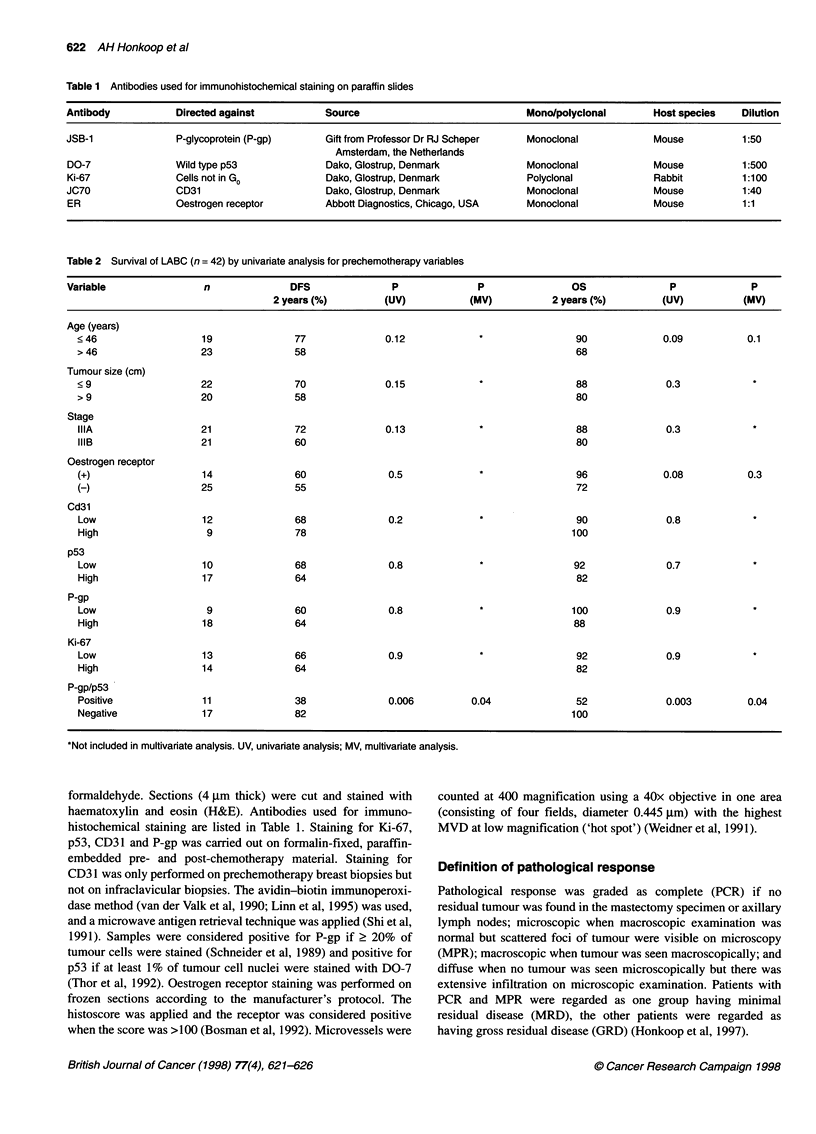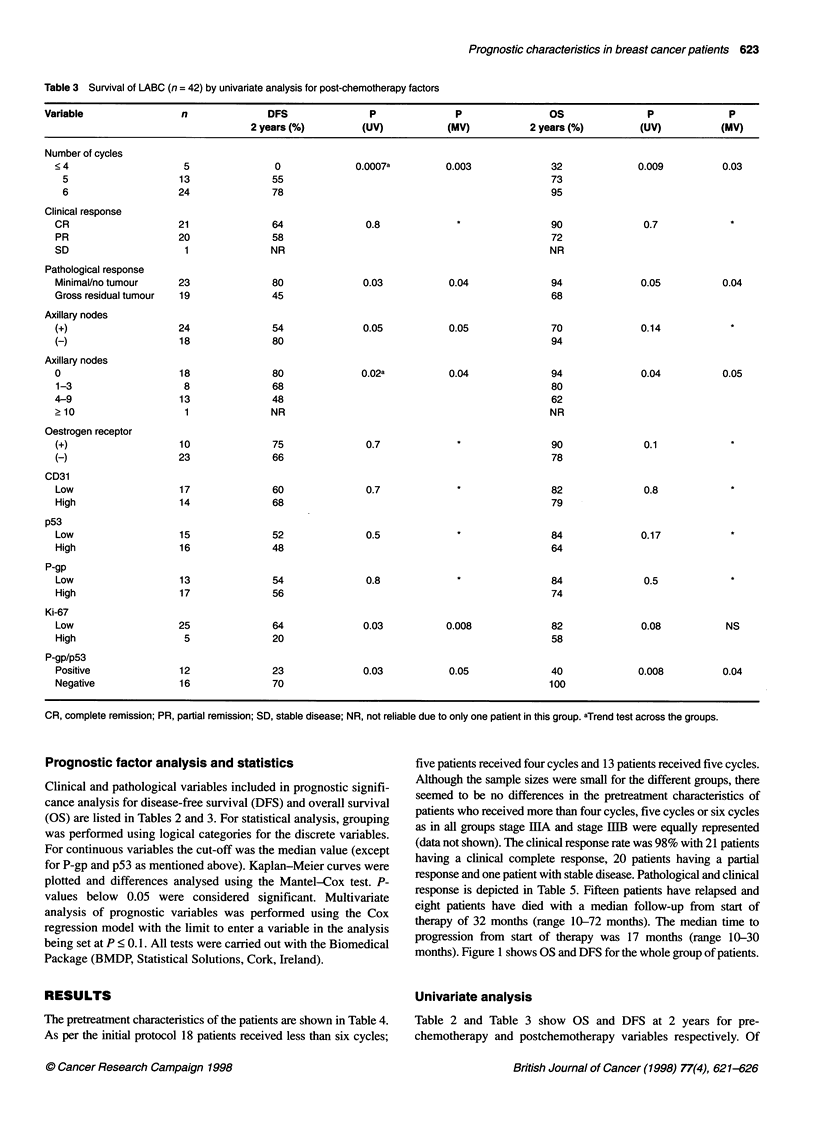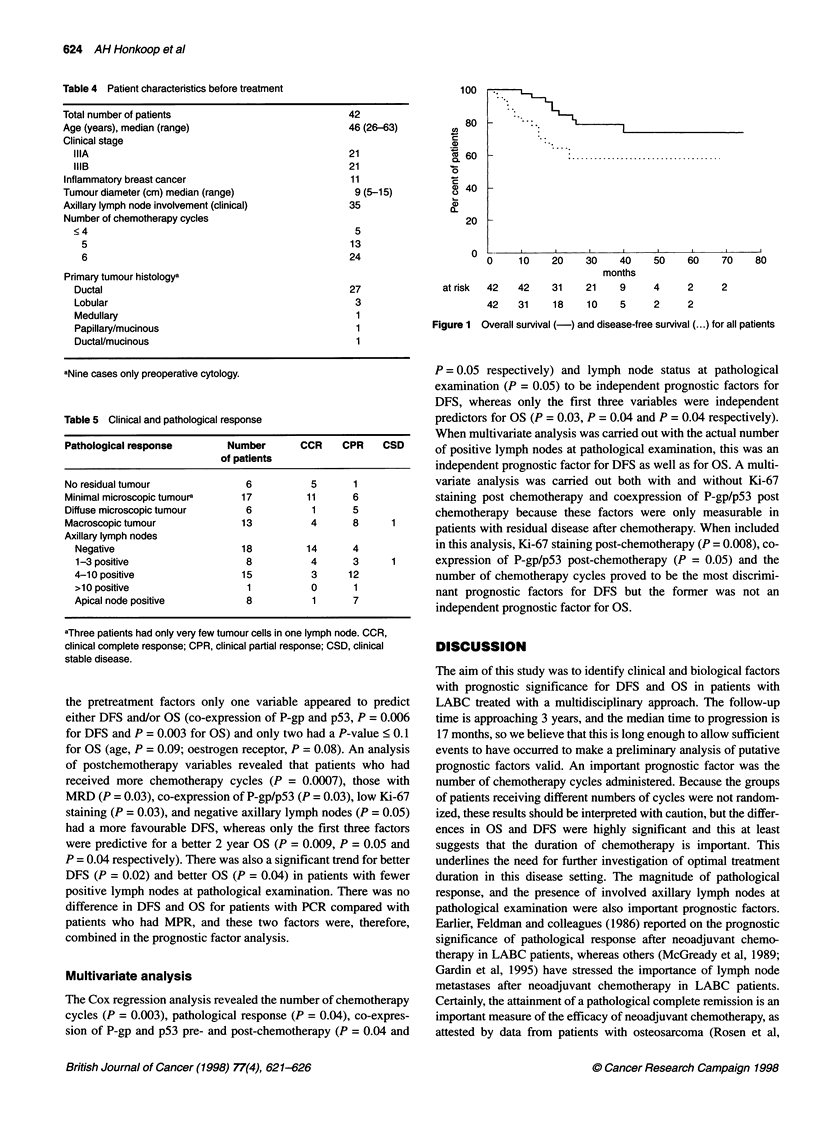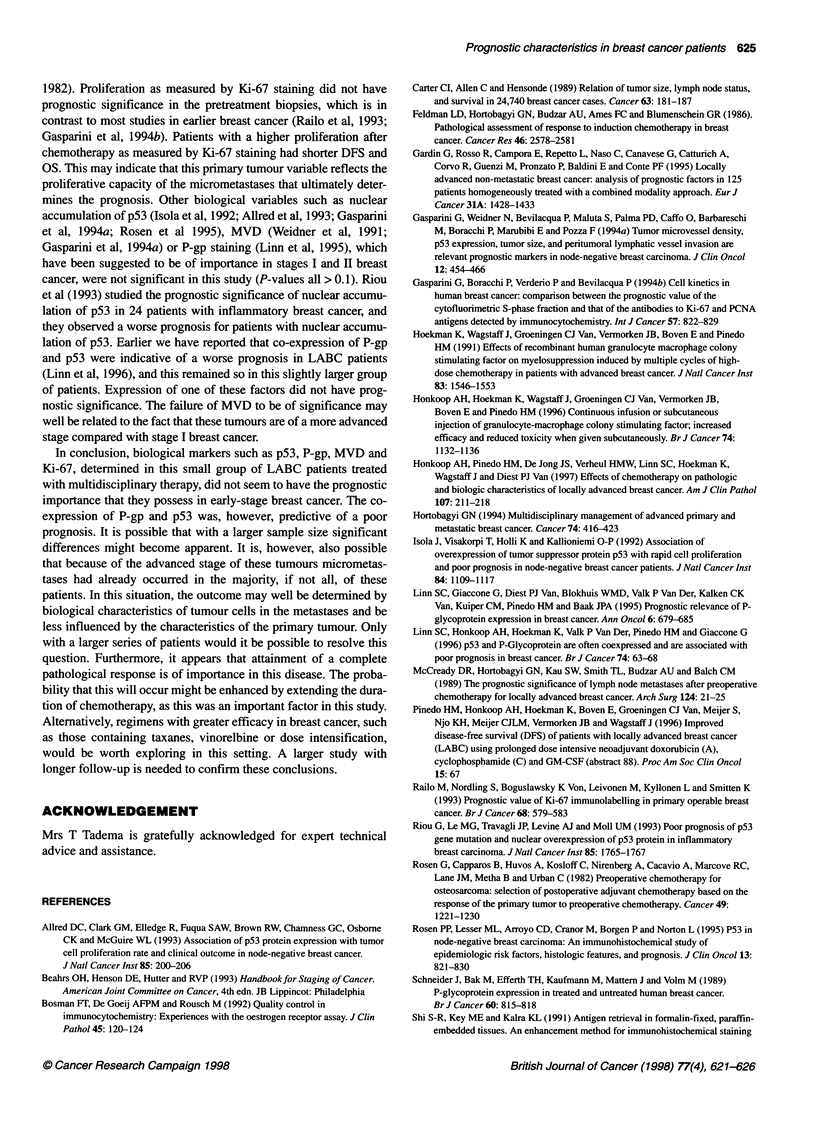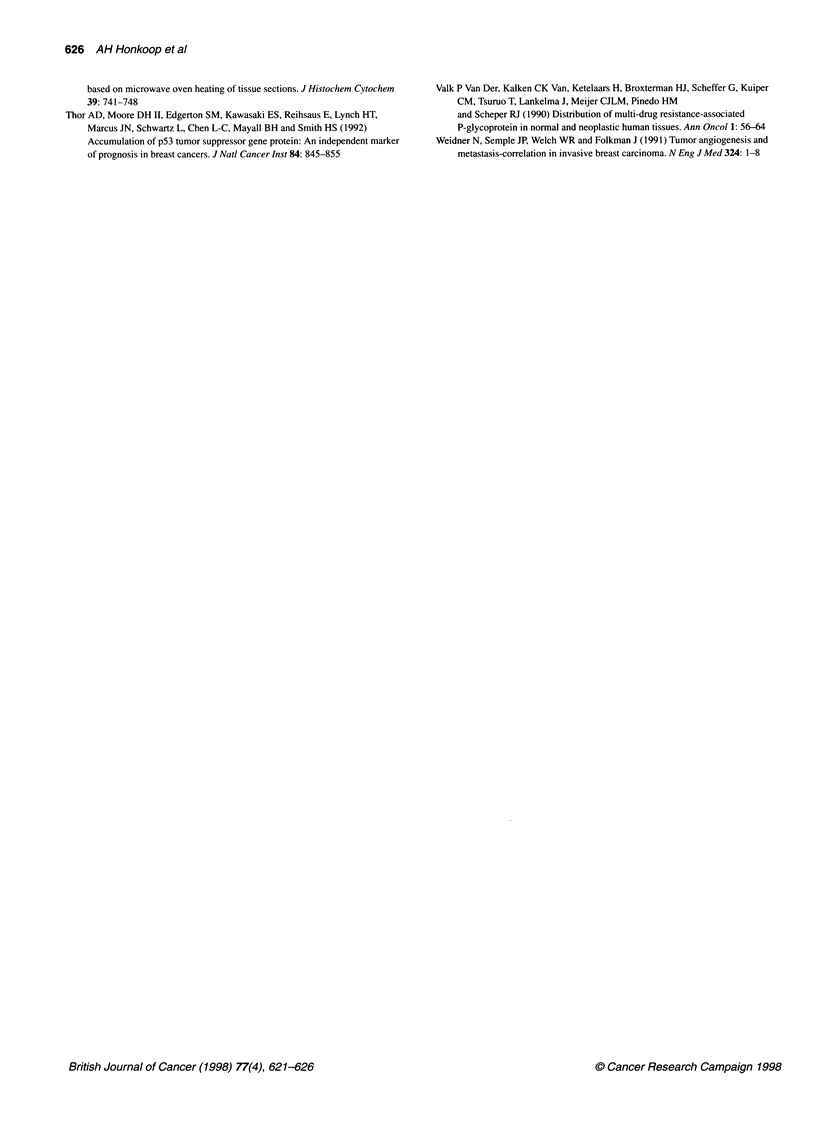# Prognostic role of clinical, pathological and biological characteristics in patients with locally advanced breast cancer.

**DOI:** 10.1038/bjc.1998.99

**Published:** 1998-02

**Authors:** A. H. Honkoop, P. J. van Diest, J. S. de Jong, S. C. Linn, G. Giaccone, K. Hoekman, J. Wagstaff, H. M. Pinedo

**Affiliations:** Department of Medical Oncology, University Hospital Vrije Universiteit, Amsterdam, The Netherlands.

## Abstract

Forty-two patients with clinical stage IIIA or IIIB breast cancer were treated with neoadjuvant chemotherapy followed by mastectomy and radiotherapy. The median follow-up was 32 months (range 10-72 months) and the median time to progression was 17 months (range 10-30 months). A multivariate analysis showed that a longer disease-free survival (DFS) was related to more chemotherapy cycles given (P = 0.003), a better pathological response to chemotherapy (P = 0.04) and fewer positive axillary lymph nodes (P = 0.05). A better overall survival (OS) was related to more chemotherapy cycles given (P = 0.03) and better pathological response to chemotherapy (P = 0.04). In patients with residual tumour after neoadjuvant chemotherapy, high levels of staining for Ki-67 was correlated with a worse DFS (P = 0.008). Other biological characteristics, including oestrogen receptor status, microvessel density (CD31 staining), P-glycoprotein (P-gp) staining and nuclear accumulation of p53, were not independent prognostic factors for either DFS or OS. If both P-gp and p53 were expressed, DFS and OS were worse in the uni- and multivariate analysis. The preliminary results of this phase II study suggest that coexpression of P-gp/p53 and a high level of staining for Ki-67 after chemotherapy are associated with a worse prognosis, and that prolonged neoadjuvant chemotherapy and the attainment of a pathological complete remission are important factors in determining outcome for patients with this disease.


British Journal of Cancer (1998) 77(4), 621–626
© 1998 Cancer Research Campaign

### Prognostic role of clinical, pathological and biological characteristics in patients with locally advanced breast cancer

**AH Honkoop[1], PJ van Diest[2], JS de Jong[2], SC Linn[1], G Giaccone[1], K Hoekman[1], J Wagstaff[1] and HM Pinedo[1]**

Departments of [1]Medical Oncology and [2]Pathology, University Hospital Vrije Universiteit, Amsterdam, The Netherlands

**Summary** Forty-two patients with clinical stage IIIA or IIIB breast cancer were treated with neoadjuvant chemotherapy followed by mastectomy and radiotherapy. The median follow-up was 32 months (range 10–72 months) and the median time to progression was 17 months (range 10–30 months). A multivariate analysis showed that a longer disease-free survival (DFS) was related to more chemotherapy cycles given ($P = 0.003$), a better pathological response to chemotherapy ($P = 0.04$) and fewer positive axillary lymph nodes ($P = 0.05$). A better overall survival (OS) was related to more chemotherapy cycles given ($P = 0.03$) and better pathological response to chemotherapy ($P = 0.04$). In patients with residual tumour after neoadjuvant chemotherapy, high levels of staining for Ki-67 was correlated with a worse DFS ($P = 0.008$). Other biological characteristics, including oestrogen receptor status, microvessel density (CD31 staining), P-glycoprotein (P-gp) staining and nuclear accumulation of p53, were not independent prognostic factors for either DFS or OS. If both P-gp and p53 were expressed, DFS and OS were worse in the uni- and multivariate analysis. The preliminary results of this phase II study suggest that coexpression of P-gp/p53 and a high level of staining for Ki-67 after chemotherapy are associated with a worse prognosis, and that prolonged neoadjuvant chemotherapy and the attainment of a pathological complete remission are important factors in determining outcome for patients with this disease.

**Keywords:** prognostic factor; locally advanced breast cancer; neoadjuvant chemotherapy

Neoadjuvant chemotherapy followed by either radiotherapy, surgery or both has improved the prognosis in patients with locally advanced breast cancer (LABC) (Hortobagyi et al, 1994). The many studies performed to date are, however, different in patient population studied, local therapy applied and chemotherapy scheme used. This makes it difficult to compare results and the optimal treatment scheme still has to be established. In stage I and II breast cancer, clinical and pathological variables have prognostic significance and are used as a guide for adjuvant therapies (Carter et al, 1989). Biological characteristics such as nuclear accumulation of mutant p53, microvessel density (MVD) and tumour cell proliferation are being used increasingly to further refine our ability to predict the prognosis of patients with early breast cancer (Weidner et al, 1991; Isola et al, 1992; Allred et al, 1993; Railo et al, 1993; Gasparini et al, 1994*a*). Recently, we reported that the expression of P-glycoprotein (P-gp), may be indicative of a worse prognosis in primary stage I–II breast cancer (Linn et al, 1995). Much less is known regarding these prognostic factors in LABC. This paper reports the preliminary results of a prognostic factor analysis on a group of women treated with neoadjuvant chemotherapy for LABC, incorporating both traditional and more recently developed factors.

*Received 12 February 1997*
*Revised 1 August 1997*
*Accepted 21 August 1997*

*Correspondence to:* HM Pinedo, Department of Medical Oncology, University Hospital Vrije Universiteit, PO Box 7057, 1007 MB Amsterdam, The Netherlands

### PATIENTS AND METHODS

### Patients

Patients with stage IIIA and stage IIIB breast cancer according to the AJCC criteria (Beahrs et al, 1993) were enrolled into a study with neoadjuvant doxorubicin 90 mg $m^{-2}$ and cyclophosphamide 1000 mg $m^{-2}$ (Pinedo et al, 1996) and GM-CSF 250 μg $m^{-2}$ (Honkoop et al, 1996). As established in a previous dose-finding study, a dose reduction of 10% relative to the previous dose level was applied in cycles 2 and 4 in every patient (Hoekman et al, 1991). Initially, it was the intention to give four to six cycles, dependent upon the rapidity of achieving a clinical complete or nearly complete remission, and the toxicity for the individual patient. When the study progressed and toxicity appeared tolerable, we aimed at the administration of six cycles whenever possible. This decision was based on the fact that gross residual tumour was often observed at pathological examination of the mastectomy specimens when fewer cycles were given. In 24 patients an incisional biopsy was performed for diagnosis. In 18 patients, referred from other hospitals, other diagnostic procedures were performed: a subclavicular biopsy in nine patients and a fine-needle aspiration in another nine patients (Honkoop et al, 1997). All patients underwent mastectomy according to Madden with axillary dissection, followed by radiotherapy (4005 cGy in 15 fractions to the thoracic wall, the internal mammary nodes, the axilla and the supraclavicular fossa). No adjuvant chemotherapy or hormones were applied.

### Processing of the histological material

The fresh mastectomy specimens or biopsies were processed using standard pathological techniques with fixation in 4% buffered

**Table 1** Antibodies used for immunohistochemical staining on paraffin slides

| Antibody | Directed against | Source | Mono/polyclonal | Host species | Dilution |
|---|---|---|---|---|---|
| JSB-1 | P-glycoprotein (P-gp) | Gift from Professor Dr RJ Scheper Amsterdam, the Netherlands | Monoclonal | Mouse | 1:50 |
| DO-7 | Wild type p53 | Dako, Glostrup, Denmark | Monoclonal | Mouse | 1:500 |
| Ki-67 | Cells not in $G_0$ | Dako, Glostrup, Denmark | Polyclonal | Rabbit | 1:100 |
| JC70 | CD31 | Dako, Glostrup, Denmark | Monoclonal | Mouse | 1:40 |
| ER | Oestrogen receptor | Abbott Diagnostics, Chicago, USA | Monoclonal | Mouse | 1:1 |

**Table 2** Survival of LABC ($n$ = 42) by univariate analysis for prechemotherapy variables

| Variable | *n* | DFS 2 years (%) | P (UV) | P (MV) | OS 2 years (%) | P (UV) | P (MV) |
|---|---|---|---|---|---|---|---|
| Age (years) | | | | | | | |
| ≤ 46 | 19 | 77 | 0.12 | * | 90 | 0.09 | 0.1 |
| > 46 | 23 | 58 | | | 68 | | |
| Tumour size (cm) | | | | | | | |
| ≤ 9 | 22 | 70 | 0.15 | * | 88 | 0.3 | * |
| > 9 | 20 | 58 | | | 80 | | |
| Stage | | | | | | | |
| IIIA | 21 | 72 | 0.13 | * | 88 | 0.3 | * |
| IIIB | 21 | 60 | | | 80 | | |
| Oestrogen receptor | | | | | | | |
| (+) | 14 | 60 | 0.5 | * | 96 | 0.08 | 0.3 |
| (–) | 25 | 55 | | | 72 | | |
| Cd31 | | | | | | | |
| Low | 12 | 68 | 0.2 | * | 90 | 0.8 | * |
| High | 9 | 78 | | | 100 | | |
| p53 | | | | | | | |
| Low | 10 | 68 | 0.8 | * | 92 | 0.7 | * |
| High | 17 | 64 | | | 82 | | |
| P-gp | | | | | | | |
| Low | 9 | 60 | 0.8 | * | 100 | 0.9 | * |
| High | 18 | 64 | | | 88 | | |
| Ki-67 | | | | | | | |
| Low | 13 | 66 | 0.9 | * | 92 | 0.9 | * |
| High | 14 | 64 | | | 82 | | |
| P-gp/p53 | | | | | | | |
| Positive | 11 | 38 | 0.006 | 0.04 | 52 | 0.003 | 0.04 |
| Negative | 17 | 82 | | | 100 | | |

*Not included in multivariate analysis. UV, univariate analysis; MV, multivariate analysis.

formaldehyde. Sections (4 µm thick) were cut and stained with haematoxylin and eosin (H&E). Antibodies used for immunohistochemical staining are listed in Table 1. Staining for Ki-67, p53, CD31 and P-gp was carried out on formalin-fixed, paraffin-embedded pre- and post-chemotherapy material. Staining for CD31 was only performed on prechemotherapy breast biopsies but not on infraclavicular biopsies. The avidin–biotin immunoperoxidase method (van der Valk et al, 1990; Linn et al, 1995) was used, and a microwave antigen retrieval technique was applied (Shi et al, 1991). Samples were considered positive for P-gp if ≥ 20% of tumour cells were stained (Schneider et al, 1989) and positive for p53 if at least 1% of tumour cell nuclei were stained with DO-7 (Thor et al, 1992). Oestrogen receptor staining was performed on frozen sections according to the manufacturer's protocol. The histoscore was applied and the receptor was considered positive when the score was >100 (Bosman et al, 1992). Microvessels were counted at 400 magnification using a 40× objective in one area (consisting of four fields, diameter 0.445 µm) with the highest MVD at low magnification ('hot spot') (Weidner et al, 1991).

### Definition of pathological response

Pathological response was graded as complete (PCR) if no residual tumour was found in the mastectomy specimen or axillary lymph nodes; microscopic when macroscopic examination was normal but scattered foci of tumour were visible on microscopy (MPR); macroscopic when tumour was seen macroscopically; and diffuse when no tumour was seen microscopically but there was extensive infiltration on microscopic examination. Patients with PCR and MPR were regarded as one group having minimal residual disease (MRD), the other patients were regarded as having gross residual disease (GRD) (Honkoop et al, 1997).

© Cancer Research Campaign 1998

**Table 3** Survival of LABC ($n$ = 42) by univariate analysis for post-chemotherapy factors

| Variable | $n$ | DFS 2 years (%) | P (UV) | P (MV) | OS 2 years (%) | P (UV) | P (MV) |
|---|---|---|---|---|---|---|---|
| Number of cycles | | | | | | | |
| ≤ 4 | 5 | 0 | 0.0007[a] | 0.003 | 32 | 0.009 | 0.03 |
| 5 | 13 | 55 | | | 73 | | |
| 6 | 24 | 78 | | | 95 | | |
| Clinical response | | | | | | | |
| CR | 21 | 64 | 0.8 | * | 90 | 0.7 | * |
| PR | 20 | 58 | | | 72 | | |
| SD | 1 | NR | | | NR | | |
| Pathological response | | | | | | | |
| Minimal/no tumour | 23 | 80 | 0.03 | 0.04 | 94 | 0.05 | 0.04 |
| Gross residual tumour | 19 | 45 | | | 68 | | |
| Axillary nodes | | | | | | | |
| (+) | 24 | 54 | 0.05 | 0.05 | 70 | 0.14 | * |
| (–) | 18 | 80 | | | 94 | | |
| Axillary nodes | | | | | | | |
| 0 | 18 | 80 | 0.02[a] | 0.04 | 94 | 0.04 | 0.05 |
| 1–3 | 8 | 68 | | | 80 | | |
| 4–9 | 13 | 48 | | | 62 | | |
| ≥ 10 | 1 | NR | | | NR | | |
| Oestrogen receptor | | | | | | | |
| (+) | 10 | 75 | 0.7 | * | 90 | 0.1 | * |
| (–) | 23 | 66 | | | 78 | | |
| CD31 | | | | | | | |
| Low | 17 | 60 | 0.7 | * | 82 | 0.8 | * |
| High | 14 | 68 | | | 79 | | |
| p53 | | | | | | | |
| Low | 15 | 52 | 0.5 | * | 84 | 0.17 | * |
| High | 16 | 48 | | | 64 | | |
| P-gp | | | | | | | |
| Low | 13 | 54 | 0.8 | * | 84 | 0.5 | * |
| High | 17 | 56 | | | 74 | | |
| Ki-67 | | | | | | | |
| Low | 25 | 64 | 0.03 | 0.008 | 82 | 0.08 | NS |
| High | 5 | 20 | | | 58 | | |
| P-gp/p53 | | | | | | | |
| Positive | 12 | 23 | 0.03 | 0.05 | 40 | 0.008 | 0.04 |
| Negative | 16 | 70 | | | 100 | | |

CR, complete remission; PR, partial remission; SD, stable disease; NR, not reliable due to only one patient in this group. [a]Trend test across the groups.

### Prognostic factor analysis and statistics

Clinical and pathological variables included in prognostic significance analysis for disease-free survival (DFS) and overall survival (OS) are listed in Tables 2 and 3. For statistical analysis, grouping was performed using logical categories for the discrete variables. For continuous variables the cut-off was the median value (except for P-gp and p53 as mentioned above). Kaplan–Meier curves were plotted and differences analysed using the Mantel–Cox test. $P$-values below 0.05 were considered significant. Multivariate analysis of prognostic variables was performed using the Cox regression model with the limit to enter a variable in the analysis being set at $P \leq 0.1$. All tests were carried out with the Biomedical Package (BMDP, Statistical Solutions, Cork, Ireland).

### RESULTS

The pretreatment characteristics of the patients are shown in Table 4. As per the initial protocol 18 patients received less than six cycles; five patients received four cycles and 13 patients received five cycles. Although the sample sizes were small for the different groups, there seemed to be no differences in the pretreatment characteristics of patients who received more than four cycles, five cycles or six cycles as in all groups stage IIIA and stage IIIB were equally represented (data not shown). The clinical response rate was 98% with 21 patients having a clinical complete response, 20 patients having a partial response and one patient with stable disease. Pathological and clinical response is depicted in Table 5. Fifteen patients have relapsed and eight patients have died with a median follow-up from start of therapy of 32 months (range 10–72 months). The median time to progression from start of therapy was 17 months (range 10–30 months). Figure 1 shows OS and DFS for the whole group of patients.

### Univariate analysis

Table 2 and Table 3 show OS and DFS at 2 years for pre-chemotherapy and postchemotherapy variables respectively. Of

© Cancer Research Campaign 1998

**Table 4** Patient characteristics before treatment

| | |
|---|---|
| Total number of patients | 42 |
| Age (years), median (range) | 46 (26–63) |
| Clinical stage | |
| IIIA | 21 |
| IIIB | 21 |
| Inflammatory breast cancer | 11 |
| Tumour diameter (cm) median (range) | 9 (5–15) |
| Axillary lymph node involvement (clinical) | 35 |
| Number of chemotherapy cycles | |
| ≤4 | 5 |
| 5 | 13 |
| 6 | 24 |
| Primary tumour histology[a] | |
| Ductal | 27 |
| Lobular | 3 |
| Medullary | 1 |
| Papillary/mucinous | 1 |
| Ductal/mucinous | 1 |

[a]Nine cases only preoperative cytology.

**Table 5** Clinical and pathological response

| Pathological response | Number of patients | CCR | CPR | CSD |
|---|---|---|---|---|
| No residual tumour | 6 | 5 | 1 | |
| Minimal microscopic tumour[a] | 17 | 11 | 6 | |
| Diffuse microscopic tumour | 6 | 1 | 5 | |
| Macroscopic tumour | 13 | 4 | 8 | 1 |
| Axillary lymph nodes | | | | |
| Negative | 18 | 14 | 4 | |
| 1–3 positive | 8 | 4 | 3 | 1 |
| 4–10 positive | 15 | 3 | 12 | |
| >10 positive | 1 | 0 | 1 | |
| Apical node positive | 8 | 1 | 7 | |

[a]Three patients had only very few tumour cells in one lymph node. CCR, clinical complete response; CPR, clinical partial response; CSD, clinical stable disease.

the pretreatment factors only one variable appeared to predict either DFS and/or OS (co-expression of P-gp and p53, $P = 0.006$ for DFS and $P = 0.003$ for OS) and only two had a $P$-value $\leq 0.1$ for OS (age, $P = 0.09$; oestrogen receptor, $P = 0.08$). An analysis of postchemotherapy variables revealed that patients who had received more chemotherapy cycles ($P = 0.0007$), those with MRD ($P = 0.03$), co-expression of P-gp/p53 ($P = 0.03$), low Ki-67 staining ($P = 0.03$), and negative axillary lymph nodes ($P = 0.05$) had a more favourable DFS, whereas only the first three factors were predictive for a better 2 year OS ($P = 0.009$, $P = 0.05$ and $P = 0.04$ respectively). There was also a significant trend for better DFS ($P = 0.02$) and better OS ($P = 0.04$) in patients with fewer positive lymph nodes at pathological examination. There was no difference in DFS and OS for patients with PCR compared with patients who had MPR, and these two factors were, therefore, combined in the prognostic factor analysis.

### Multivariate analysis

The Cox regression analysis revealed the number of chemotherapy cycles ($P = 0.003$), pathological response ($P = 0.04$), co-expression of P-gp and p53 pre- and post-chemotherapy ($P = 0.04$ and $P = 0.05$ respectively) and lymph node status at pathological examination ($P = 0.05$) to be independent prognostic factors for DFS, whereas only the first three variables were independent predictors for OS ($P = 0.03$, $P = 0.04$ and $P = 0.04$ respectively). When multivariate analysis was carried out with the actual number of positive lymph nodes at pathological examination, this was an independent prognostic factor for DFS as well as for OS. A multivariate analysis was carried out both with and without Ki-67 staining post chemotherapy and coexpression of P-gp/p53 post chemotherapy because these factors were only measurable in patients with residual disease after chemotherapy. When included in this analysis, Ki-67 staining post-chemotherapy ($P = 0.008$), co-expression of P-gp/p53 post-chemotherapy ($P = 0.05$) and the number of chemotherapy cycles proved to be the most discriminant prognostic factors for DFS but the former was not an independent prognostic factor for OS.

**Figure 1** Overall survival (—) and disease-free survival (...) for all patients

### DISCUSSION

The aim of this study was to identify clinical and biological factors with prognostic significance for DFS and OS in patients with LABC treated with a multidisciplinary approach. The follow-up time is approaching 3 years, and the median time to progression is 17 months, so we believe that this is long enough to allow sufficient events to have occurred to make a preliminary analysis of putative prognostic factors valid. An important prognostic factor was the number of chemotherapy cycles administered. Because the groups of patients receiving different numbers of cycles were not randomized, these results should be interpreted with caution, but the differences in OS and DFS were highly significant and this at least suggests that the duration of chemotherapy is important. This underlines the need for further investigation of optimal treatment duration in this disease setting. The magnitude of pathological response, and the presence of involved axillary lymph nodes at pathological examination were also important prognostic factors. Earlier, Feldman and colleagues (1986) reported on the prognostic significance of pathological response after neoadjuvant chemotherapy in LABC patients, whereas others (McGready et al, 1989; Gardin et al, 1995) have stressed the importance of lymph node metastases after neoadjuvant chemotherapy in LABC patients. Certainly, the attainment of a pathological complete remission is an important measure of the efficacy of neoadjuvant chemotherapy, as attested by data from patients with osteosarcoma (Rosen et al,

© Cancer Research Campaign 1998

1982). Proliferation as measured by Ki-67 staining did not have prognostic significance in the pretreatment biopsies, which is in contrast to most studies in earlier breast cancer (Railo et al, 1993; Gasparini et al, 1994*b*). Patients with a higher proliferation after chemotherapy as measured by Ki-67 staining had shorter DFS and OS. This may indicate that this primary tumour variable reflects the proliferative capacity of the micrometastases that ultimately determines the prognosis. Other biological variables such as nuclear accumulation of p53 (Isola et al, 1992; Allred et al, 1993; Gasparini et al, 1994*a*; Rosen et al 1995), MVD (Weidner et al, 1991; Gasparini et al, 1994*a*) or P-gp staining (Linn et al, 1995), which have been suggested to be of importance in stages I and II breast cancer, were not significant in this study ($P$-values all > 0.1). Riou et al (1993) studied the prognostic significance of nuclear accumulation of p53 in 24 patients with inflammatory breast cancer, and they observed a worse prognosis for patients with nuclear accumulation of p53. Earlier we have reported that co-expression of P-gp and p53 were indicative of a worse prognosis in LABC patients (Linn et al, 1996), and this remained so in this slightly larger group of patients. Expression of one of these factors did not have prognostic significance. The failure of MVD to be of significance may well be related to the fact that these tumours are of a more advanced stage compared with stage I breast cancer.

In conclusion, biological markers such as p53, P-gp, MVD and Ki-67, determined in this small group of LABC patients treated with multidisciplinary therapy, did not seem to have the prognostic importance that they possess in early-stage breast cancer. The co-expression of P-gp and p53 was, however, predictive of a poor prognosis. It is possible that with a larger sample size significant differences might become apparent. It is, however, also possible that because of the advanced stage of these tumours micrometastases had already occurred in the majority, if not all, of these patients. In this situation, the outcome may well be determined by biological characteristics of tumour cells in the metastases and be less influenced by the characteristics of the primary tumour. Only with a larger series of patients would it be possible to resolve this question. Furthermore, it appears that attainment of a complete pathological response is of importance in this disease. The probability that this will occur might be enhanced by extending the duration of chemotherapy, as this was an important factor in this study. Alternatively, regimens with greater efficacy in breast cancer, such as those containing taxanes, vinorelbine or dose intensification, would be worth exploring in this setting. A larger study with longer follow-up is needed to confirm these conclusions.

### ACKNOWLEDGEMENT

Mrs T Tadema is gratefully acknowledged for expert technical advice and assistance.

### REFERENCES

Allred DC, Clark GM, Elledge R, Fuqua SAW, Brown RW, Chamness GC, Osborne CK and McGuire WL (1993) Association of p53 protein expression with tumor cell proliferation rate and clinical outcome in node-negative breast cancer. *J Natl Cancer Inst* **85**: 200–206

Beahrs OH, Henson DE, Hutter and RVP (1993) *Handbook for Staging of Cancer. American Joint Committee on Cancer*, 4th edn. JB Lippincot: Philadelphia

Bosman FT, De Goeij AFPM and Rousch M (1992) Quality control in immunocytochemistry: Experiences with the oestrogen receptor assay. *J Clin Pathol* **45**: 120–124

Carter CI, Allen C and Hensonde (1989) Relation of tumor size, lymph node status, and survival in 24,740 breast cancer cases. *Cancer* **63**: 181–187

Feldman LD, Hortobagyi GN, Budzar AU, Ames FC and Blumenschein GR (1986). Pathological assessment of response to induction chemotherapy in breast cancer. *Cancer Res* **46**: 2578–2581

Gardin G, Rosso R, Campora E, Repetto L, Naso C, Canavese G, Catturich A, Corvo R, Guenzi M, Pronzato P, Baldini E and Conte PF (1995) Locally advanced non-metastatic breast cancer: analysis of prognostic factors in 125 patients homogeneously treated with a combined modality approach. *Eur J Cancer* **31A**: 1428–1433

Gasparini G, Weidner N, Bevilacqua P, Maluta S, Palma PD, Caffo O, Barbareschi M, Boracchi P, Marubibi E and Pozza F (1994*a*) Tumor microvessel density, p53 expression, tumor size, and peritumoral lymphatic vessel invasion are relevant prognostic markers in node-negative breast carcinoma. *J Clin Oncol* **12**: 454–466

Gasparini G, Boracchi P, Verderio P and Bevilacqua P (1994*b*) Cell kinetics in human breast cancer: comparison between the prognostic value of the cytofluorimetric S-phase fraction and that of the antibodies to Ki-67 and PCNA antigens detected by immunocytochemistry. *Int J Cancer* **57**: 822–829

Hoekman K, Wagstaff J, Groeningen CJ Van, Vermorken JB, Boven E and Pinedo HM (1991) Effects of recombinant human granulocyte macrophage colony stimulating factor on myelosuppression induced by multiple cycles of high-dose chemotherapy in patients with advanced breast cancer. *J Natl Cancer Inst* **83**: 1546–1553

Honkoop AH, Hoekman K, Wagstaff J, Groeningen CJ Van, Vermorken JB, Boven E and Pinedo HM (1996) Continuous infusion or subcutaneous injection of granulocyte-macrophage colony stimulating factor; increased efficacy and reduced toxicity when given subcutaneously. *Br J Cancer* **74**: 1132–1136

Honkoop AH, Pinedo HM, De Jong JS, Verheul HMW, Linn SC, Hoekman K, Wagstaff J and Diest PJ Van (1997) Effects of chemotherapy on pathologic and biologic characteristics of locally advanced breast cancer. *Am J Clin Pathol* **107**: 211–218

Hortobagyi GN (1994) Multidisciplinary management of advanced primary and metastatic breast cancer. *Cancer* **74**: 416–423

Isola J, Visakorpi T, Holli K and Kallioniemi O-P (1992) Association of overexpression of tumor suppressor protein p53 with rapid cell proliferation and poor prognosis in node-negative breast cancer patients. *J Natl Cancer Inst* **84**: 1109–1117

Linn SC, Giaccone G, Diest PJ Van, Blokhuis WMD, Valk P Van Der, Kalken CK Van, Kuiper CM, Pinedo HM and Baak JPA (1995) Prognostic relevance of P-glycoprotein expression in breast cancer. *Ann Oncol* **6**: 679–685

Linn SC, Honkoop AH, Hoekman K, Valk P Van Der, Pinedo HM and Giaccone G (1996) p53 and P-Glycoprotein are often coexpressed and are associated with poor prognosis in breast cancer. *Br J Cancer* **74**: 63–68

McCready DR, Hortobagyi GN, Kau SW, Smith TL, Budzar AU and Balch CM (1989) The prognostic significance of lymph node metastases after preoperative chemotherapy for locally advanced breast cancer. *Arch Surg* **124**: 21–25

Pinedo HM, Honkoop AH, Hoekman K, Boven E, Groeningen CJ Van, Meijer S, Njo KH, Meijer CJLM, Vermorken JB and Wagstaff J (1996) Improved disease-free survival (DFS) of patients with locally advanced breast cancer (LABC) using prolonged dose intensive neoadjuvant doxorubicin (A), cyclophosphamide (C) and GM-CSF (abstract 88). *Proc Am Soc Clin Oncol* **15**: 67

Railo M, Nordling S, Boguslawsky K Von, Leivonen M, Kyllonen L and Smitten K (1993) Prognostic value of Ki-67 immunolabelling in primary operable breast cancer. *Br J Cancer* **68**: 579–583

Riou G, Le MG, Travagli JP, Levine AJ and Moll UM (1993) Poor prognosis of p53 gene mutation and nuclear overexpression of p53 protein in inflammatory breast carcinoma. *J Natl Cancer Inst* **85**: 1765–1767

Rosen G, Capparos B, Huvos A, Kosloff C, Nirenberg A, Cacavio A, Marcove RC, Lane JM, Metha B and Urban C (1982) Preoperative chemotherapy for osteosarcoma: selection of postoperative adjuvant chemotherapy based on the response of the primary tumor to preoperative chemotherapy. *Cancer* **49**: 1221–1230

Rosen PP, Lesser ML, Arroyo CD, Cranor M, Borgen P and Norton L (1995) P53 in node-negative breast carcinoma: An immunohistochemical study of epidemiologic risk factors, histologic features, and prognosis. *J Clin Oncol* **13**: 821–830

Schneider J, Bak M, Efferth TH, Kaufmann M, Mattern J and Volm M (1989) P-glycoprotein expression in treated and untreated human breast cancer. *Br J Cancer* **60**: 815–818

Shi S-R, Key ME and Kalra KL (1991) Antigen retrieval in formalin-fixed, paraffin-embedded tissues. An enhancement method for immunohistochemical staining

© Cancer Research Campaign 1998

based on microwave oven heating of tissue sections. *J Histochem Cytochem* **39**: 741–748

Thor AD, Moore DH II, Edgerton SM, Kawasaki ES, Reihsaus E, Lynch HT, Marcus JN, Schwartz L, Chen L-C, Mayall BH and Smith HS (1992) Accumulation of p53 tumor suppressor gene protein: An independent marker of prognosis in breast cancers. *J Natl Cancer Inst* **84**: 845–855

Valk P Van Der, Kalken CK Van, Ketelaars H, Broxterman HJ, Scheffer G, Kuiper CM, Tsuruo T, Lankelma J, Meijer CJLM, Pinedo HM and Scheper RJ (1990) Distribution of multi-drug resistance-associated P-glycoprotein in normal and neoplastic human tissues. *Ann Oncol* **1**: 56–64

Weidner N, Semple JP, Welch WR and Folkman J (1991) Tumor angiogenesis and metastasis-correlation in invasive breast carcinoma. *N Eng J Med* **324**: 1–8

© Cancer Research Campaign 1998